# Adenosine Deaminase and Systemic Immune Inflammatory Index—A Biomarker Duet Signature of Pulmonary Tuberculosis Severity

**DOI:** 10.3390/medicina61061096

**Published:** 2025-06-17

**Authors:** Ioan Anton Arghir, Oana Cristina Arghir, Marina Ruxandra Otelea, Iulia Tania Andronache, Ileana Ion

**Affiliations:** 1 Doctoral School of Medicine, “Ovidius” University of Constanta, 1 University Alley, 900470 Constanta, Romania; ionut_arghir93@yahoo.com (I.A.A.); arghir_oana@yahoo.com (O.C.A.); ileana_ion58@yahoo.com (I.I.); 2 Clinical Internal 4th Department, Medicine Faculty, “Ovidius” University of Constanta, 1 University Alley, 900470 Constanta, Romania; 3 Constanta Clinical Pneumology Hospital, 40 Sentinelei Str., 900002 Constanta, Romania; 4 Occupational Diseases Department, Medicine Faculty, “Carol Davila” University of Medicine and Pharmacy, 8 Eroii Sanitari Blvd., 050474 Bucharest, Romania; 5 Occupational Department, Colentina Clinical Hospital, 19-21 Stefan cel Mare, 020125 Bucharest, Romania; 6 Internal Medicine Clinic, “Dr. Alexandru Gafencu” Military Emergency Hospital, 96 Mamaia Blvd., 900527 Constanta, Romania; andronacheiulia@gmail.com

**Keywords:** pulmonary tuberculosis, serum adenosine deaminase, systemic immune inflammatory index, biomarkers

## Abstract

*Background and Objectives*: The role of adenosine deaminase (ADA) in pulmonary tuberculosis (PTB) remains insufficiently defined in advanced forms of disease. Likewise, the systemic immune inflammatory index (SII) has not been validated in severe PTB. This 6-year prospective observational study aims to evaluate biomarker signatures of serum ADA and SII. *Materials and Methods*: According to the PTB case definition, 232 adult patients were divided into group 1, with a positive bacteriologic exam (n = 168), and group 2, without bacteriological confirmation (n = 64). ADA serum levels were compared by age, gender, nutritional status, morphologic and bacteriological pattern of PTB lesions, survival status, along with serum levels of other inflammatory biomarkers. All patients with comorbidities, interfering with the level of ADA, were excluded to avoid bias. *Results*: A total cohort of 208 PTB adults, aged 54.37 ± 14.365 years, included 156 males. The overall mortality was 11.53%. Death occurred after a mean interval of 1.63 ± 3.228 months after PTB diagnosis. ADA serum mean levels were 32.94 ± 9.146 IU/L, significantly higher in G1 (*p* = 0.002), in patients with delayed diagnosis of PTB (*p* = 0.000), with lung cavitation (*p* = 0.003), and death as a poor outcome (*p* ˂ 0.02). SII had a mean value of 1752.226 ± 2704.150, significantly increased in bacteriologically confirmed PTB cases (*p* = 0.018), delayed diagnosis (*p* = 0.002), cavitary advanced pulmonary tuberculosis (APT) (*p* = 0.002), and deceased (*p* = 0.003). Both an ADA cut-off elevated risk value of over 30 IU/L and SII of over 902 were fulfilled by 73 patients, with 2.10 higher risk of advanced PTB (*p* = 0.006) and 4.49 higher risk of mortality (*p* = 0.000). *Conclusions*: Serum ADA and SII are recommended as predictors of advanced and severe pulmonary TB. These findings indicate that ADA and SII, when elevated together, delineate a high-risk PTB phenotype with greater disease severity and early mortality. The combination offers a pragmatic, biomarker-based approach to risk stratification in PTB.

## 1. Introduction

Despite advancements in medical diagnostics, pulmonary tuberculosis (PTB) remains a life-threatening disease in the second decade of the third millennium, particularly following the COVID-19 pandemic. The World Health Organization (WHO) estimated nearly 11 million incident TB cases and approximately 1 million deaths globally for the years 2023 and 2024 [1,2]. Delayed diagnosis, especially when extensive cavitary lung disease is present, critically affects prognosis and elevates mortality risk. In 2009, MacLaren Wallace et al. were the first to define advanced pulmonary TB (PTB) as TB disease with lung cavitation on chest X-ray having positive acid-fast-bacilli on smear-positive sputum, reflecting the delayed diagnosis (DD) of tuberculosis (TB) disease and contributing to severe evolution to relapses or death, as poor outcomes [3]. Delayed diagnosis (DD) of PTB explains advanced lung lesions with cavitary extension and a highly infectious character, facilitating the spread of TB bacilli, before, during, and after the COVID-19 pandemic [4,5,6,7].

Tuberculosis demonstrates its significant clinical versatility, with multiple intermediate stages following initial contact with Mycobacterium tuberculosis (MTB). Clinically, the disease primarily manifests in two evolutionary scenarios. The first, classic presentation involves advanced cavitary lesions resulting from caseation, with microbiologically confirmed positive sputum smear and/or culture. The second scenario represents diagnostic challenges, characterized by imaging abnormalities with uncertain lesion activity, encompassing both old, healed lesions and newer, progressive lesions, alongside nonspecific clinical symptoms and an absence of bacteriological confirmation. Determining the appropriate timing to initiate anti-TB therapy in this latter patient group and evaluating the role of surrogate biomarkers indicative of active disease remain significant clinical dilemmas.

Adenosine deaminase (ADA) is an enzyme primarily produced by lymphocytes and macrophages in response to MTB antigens, having a crucial role in activating monocytes and facilitating their differentiation into macrophages, thus serving as a key marker of cellular immunity in tuberculosis [8]. MTB replication within macrophages indirectly stimulates T-cell activation and proliferation, leading to elevated ADA levels, which correlate with MTB activity [9]. Since its identification in 1978, pleural ADA has been extensively documented as a valuable biomarker for diagnosing pleural fluid etiology. However, serum ADA levels have received less attention in advanced pulmonary TB despite being a rapid, cost-effective, and reliable diagnostic tool suitable for laboratories with limited resources [10,11]. Compared to previously studied or emerging biomarkers in pulmonary tuberculosis—such as lymphokine panels or serum proteomic profiles used primarily to differentiate active disease from latent TB infection, ADA remains a compelling candidate [12,13,14]. Its applicability spans both bacteriologically confirmed and clinically diagnosed cases. While several studies have explored serum ADA levels in PTB patients versus healthy controls, there is a notable paucity of data specifically addressing its prognostic value in advanced pulmonary tuberculosis, particularly in relation to earlier disease stages. The capacity to stratify risk and anticipate poor outcomes in delayed diagnosis of PTB could offer a meaningful advancement in clinical tuberculosis management.

This prospective observational study aims to evaluate serum ADA, as a potential biomarker in adult patients with advanced and delayed pulmonary tuberculosis, with emphasis on its distribution in relation to cavitary lung lesions, bacteriological confirmation, and mortality—clinical features indicative of severe disease progression. The prognostic value of plasma ADA was examined in patients diagnosed with and treated for PTB, excluding those with comorbid conditions known to elevate ADA levels independently of tuberculosis-related immune activation. The study also seeks to determine a clinically applicable ADA cut-off value associated with advanced pulmonary tuberculosis (APT). The primary objective is to assess the utility of serum ADA in characterizing disease severity and stratifying risk in both bacteriologically confirmed and clinically diagnosed forms of PTB.

## 2. Materials and Methods

### 2.1. Ethical Approval

All eligible participants aged ≥18 years provided written informed consent prior to enrolment, procedures, and access to their demographic and medical data. The study protocol was reviewed and approved by the Institutional Ethics Committee of Constanța Clinical Pneumology Hospital (No 745/11 February 2020), and the research was conducted in accordance with the Declaration of Helsinki and national regulations governing clinical research involving human subjects.

### 2.2. Participants

Over a six-year period, from January 2020 to March 2025, a total of 232 adult patients diagnosed with pulmonary tuberculosis (PTB) were evaluated at Constanța Clinical Pneumology Hospital, undergoing routine blood tests including serum adenosine deaminase (ADA) measurement. To minimize confounding factors, patients with conditions known to elevate serum ADA—such as autoimmune disorders, chronic liver disease, lymphoproliferative diseases, empyema, malignancies, or concurrent HIV and COVID-19 infections—were excluded from the study to remove confounding bias. Following the application of the inclusion and exclusion criteria detailed below (Section 2.3), 208 eligible cases were selected.

### 2.3. Methodology

A prospective observational study was conducted over a six-year period, from 2020 to 2024, at Constanța Clinical Pneumology Hospital. A total of 232 adult patients were evaluated for suspected pulmonary tuberculosis (PTB) disease and underwent serum adenosine deaminase (ADA) assay, as well as other measurements (leukocytes, lymphocytes, neutrophils platelets count, erythrocyte sedimentation rate, C-reactive protein, fibrinogen) in the first week after admission, before anti-TB therapy started, as part of their initial evaluation. In accordance with World Health Organization (WHO) guidelines [15,16] on TB classification, patients were classified into 2 groups:

- Group 1 included 168 individuals with bacteriologically confirmed PTB cases, based on positive results by smear microscopy, culture, or molecular diagnostic assays approved by WHO (Xpert MTB/RIF).
- Group 2 comprised 64 patients, with clinically diagnosed PTB cases, because bacteriological confirmation was missing. High suspicion of TB active disease was based on a combination of clinical features, radiologic abnormalities, or suggestive histopathological evidence.

In accordance with the initial definition of advanced pulmonary tuberculosis, proposed by MacLaren Wallace et al. [3], and to emphasize the predictive relevance of serum ADA in distinguishing severe advanced disease from clinically diagnosed PTB, the study design did not incorporate a healthy control group as a comparator. The methodological focus was limited to intra-cohort stratification based on disease severity and confirmation status.

#### 2.3.1. Inclusion Criteria

Inclusion criteria included age ≥18 years, written informed consent, notified PTB active disease (new cases or relapses), imagistic assessment by computed tomography (CT) of the chest, plasmatic ADA assessment included in the routine blood tests, and 2 months follow-up for cases without bacteriological confirmation.

#### 2.3.2. Exclusion Criteria

Exclusion criteria included extrapulmonary TB disease, without pulmonary involvement, an indeterminate TB status of activity noticed, in clinically diagnosed PTB, after 2 months of anti-TB therapy, consisting in unfavorable dynamic of imagistic lung lesions and no clinical improvement, presence of any comorbidity (as HIV infection, concomitant COVID-19, malignancies, autoimmune diseases, liver diseases) or empyema complicating PTB, or any other medical condition related to increased ADA (up to 19 IU/L) in order to avoid bias.

A total of 24 patients (12 from each group) were excluded, resulting in 156 bacteriologically confirmed PTB cases (group 1) and 52 clinically diagnosed PTB cases (group 2) eligible for final analysis.

Standard evaluation included clinical, radiologic, and bacteriologic investigations, anamnestic complete data about residence, previous PTB, comorbidities, exposure to smoking, symptom onset and evolution, height, weight, body mass index (BMI), and loss in weight at admission. Underweight was defined by a BMI threshold of <18.5 kg/m^2^ [17], while nutritional risk was further assessed using the Malnutrition Universal Screening Tool (MUST) [18,19]. Pulmonary imaging included both chest X-rays and computed tomography (CT) scans to document radiologic abnormalities. Bacteriological confirmation relied on smear microscopy, culture (both liquid BACTEC MGIT 960 and solid Lowenstein–Jensen media), and rapid molecular diagnostics including Line Probe Assay (LPA), Xpert MTB/RIF, and Xpert MTB/XDR.

Case classification into new diagnoses or relapses was established according to national notification criteria, drawing on both electronic hospital records and paper-based medical charts documenting previous TB episodes. Laboratory parameters included inflammatory and immunological markers: ESR, C-reactive protein (CRP), fibrinogen, plasma ADA, and full blood counts. Derived indices were computed according to validated formulas: neutrophil-to-lymphocyte ratio (NLR), platelet-to-lymphocyte ratio (PLR), and the systemic immune inflammatory index (SII = neutrophils × platelets/lymphocytes). Assessment of gamma interferon releasing assay (IGRA) was performed by QuantiFERON test (TB Gold Plus) in patients that have not undergone previous tuberculin skin testing.

### 2.4. Data Analysis Methodology

All statistical analyses were performed using IBM SPSS Statistics, version 20. All patients′ characteristics included in the database were described. Continuous variables were assessed for normality using the Shapiro–Wilk test and assessed visually through histograms and plots. Data are reported as mean ± standard deviation (std dev) for normally distributed variables and median with interquartile range (IQR) for non-normally distributed variables. Predictive biomarkers of PTB-related activity, severity, and mortality were assessed in relation to age, sex, nutritional status (assessed via body mass index), the morphological pattern of pulmonary lesions (such as cavitary or infiltrative forms), and bacteriological status. Additionally, ADA values were compared with several immunological and inflammatory biomarkers, including QuantiFERON-TB Gold Plus, total leukocyte and lymphocyte counts, neutrophil-to-lymphocyte ratio (NLR), platelet-to-lymphocyte ratio (PLR), systemic immune inflammation index (SII, calculated as neutrophils × platelets/lymphocytes), erythrocyte sedimentation rate (ESR), C-reactive protein (CRP), and fibrinogen.

Comparisons between two independent groups used either independent samples *t*-test, when both groups showed normal distribution and homogeneity of variances (verified by Levene’s test), or Mann–Whitney U test, when the assumption of normality was not met. The ANOVA analysis was used to compare quantitative variables in cases. Paired samples T test was used to compare means. An ADA value equal to or below 19 IU/L was considered normal and above 19 IU/L was considered elevated. Correlations between continuous variables were evaluated by Spearman rank correlation coefficient, as normality assumptions were not consistently met. To explore the diagnostic plasmatic biomarker levels, receiver operating characteristic (ROC) curve analysis was performed, using bacteriological confirmation and survival outcomes as the binary outcome variable. The area under the curve (AUC) greater than 6 was accepted and used to evaluate the discriminatory power of ADA and other biomarkers. The optimal cut-off value at high risk was determined according to sensitivity and specificity, revealed by ROC analysis, and it was assessed in terms of both predictive positive and negative value, among bacteriologically and clinically diagnosed PTB cases, among survivors and deceased. All tests were two-tailed, and a *p*-value < 0.05 was considered indicative of statistical significance level.

## 3. Results

The study cohort included 208 adults, with a mean age of 54.4 ± 14.4 years (limits: 18–82 years), diagnosed with pulmonary tuberculosis (PTB). Table 1 shows the descriptive characteristics of the study cases, divided by their status of confirmation (bacteriologically or clinically) into group 1 (n = 156), with smear microscopy and/or culture positivity, and group 2 (n = 52), with negative smears and cultures (Table 1).

New cases (NC) of PTB (n = 156; 208), accounting for 75% of the study population, were more frequent (96.15%) in group 2, compared to group 1, with more relapses (*p* = 0.000).

Molecular tests with positive results were reported in 148 patients from group 1. The prevalence of a drug-resistant (DR) pattern of 11.5% was revealed by genotypic methods and/or phenotypic drug sensitivity testing (DST). In more than a half of relapses (n = 10/18; 55.55%), we identified different patterns of resistance against anti-TB drugs, including four cases with monoresistance against to isoniazid (H), three cases with resistance against isoniazid and ethionamide, four cases with rifampicin resistance (RR-TB), and seven cases with a multidrug-resistant (MDR) pattern (against both HR, in association with other first-line drugs such as streptomycin, ethambutol, and pyrazinamide). Most cases, from group 1, were drug-sensitive (DS) forms of PTB (n = 138/156; 89.10%).

Death was reported, during hospitalization, in 24 patients (11.63%), 91.66% of whom were from group 1 (*p* = 0.046). PTB diagnosis preceded death by 50.50 ± 4.35 days (*p* = 0.416), and symptoms preceded PTB diagnosis by 3.30 ± 2.862 months, with a prolonged interval being observed predominantly in group 1 (*p* = 0.027) (Table 1).

The distribution of mean age, by gender, revealed similarities between men and women (54.61 ± 16.532 years versus 54.29 ± 13.583 years; F = 0.020; *p* = 0.887). The mean age of bacteriologically confirmed PTB cases was significantly lower, at the moment of diagnosis [52.81 ± 14.131 years (limits: 18–82 years)], compared to those with clinically diagnosed PTB [59.04 ± 14.181 years (limits: 23–81 years)] (F = 7.554; *p* = 0.007), but, at the moment of death, there was no difference between groups (F = 2.330; *p* = 0.141) (Table 1).

The nutritional status of patients, assessed by the MUST score, which incorporates information about height and the current and past weight, revealed significant higher scores distributed in patients from group 1 (eta = 0.328; χ^2^ =27.801; *p* = 0.000) (Table 1). The high risk of malnutrition was determined in 73.01% of patients from group 1 (n = 114/156; eta = 0.306; χ^2^ = 23.347; *p* = 0.000) (Table 1), 73.65% of patients with delayed diagnosis (n = 109/148; 73.65%; eta = 0.300; χ^2^ =21.234; *p* = 0.000), and 79.16% of deceased (n = 19/24; eta = 0.099; χ^2^ = 3.289; *p* ˂ 0.2). Clinical cachexia was documented in 67 PTB patients, associated with bacteriologically confirmed cases (n = 59/156; 37.82%; χ^2^ =7.954; *p* = 0.005) and death (n = 15/24; 62.5%; χ^2^ =9.837; *p* = 0.002).

Table 2 presents a comparison of the biomarkers measured in the two study groups at baseline (Table 2).

Almost a quarter of cases were assessed by QuantiFERON TB Gold Plus (QIAGEN, Hilden, Germany) (n = 51; 24.51%), and 80.39% of them (n = 41/51) had positive results, including two conversions from negative to positive in 6 months of consecutive determinations. Tuberculin skin testing was not performed before IGRA. The distribution of mean values among positive reactors was significantly statistically higher in group 1 versus 2 for every peptide antigen (Table 2).

**Figure 1:**
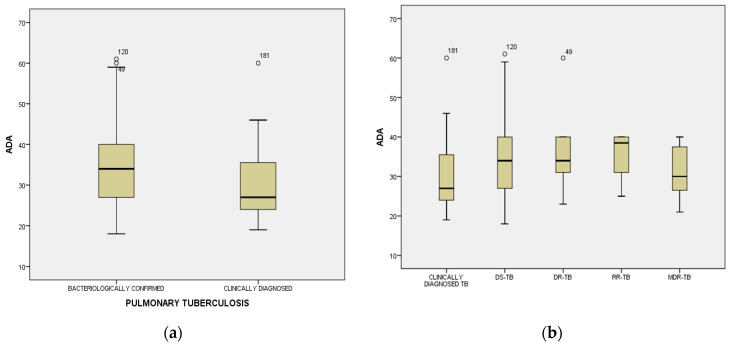
Distribution mean values of adenosine deaminase (ADA) among cases divided by World Health Organization definitions criteria [7,8]: clinically diagnosed and bacteriologically confirmed TB (**a**), including profile of drug sensitivity (DS), drug resistance (DR), rifampicin resistance (RR), and multidrug resistance (MDR) (**b**).

ADA mean level (32.94 ± 9.14 IU/L), detected in serum samples, collected from cases, was not influenced by age (F = 1.096; *p* = 0.344), gender (33.46 ± 8.755 IU/L in males, versus 31.44 ± 10.118 IU/L in females; F = 1.953; *p* = 0.164), or by residence (32.96 ± 9.245 IU/L in 113 patients with urban residence, versus 32.91 ± 9.075 IU/L in 95 patients with rural residence; F = 0.002; *p* = 0.963). Elevated average serum levels of ADA were significantly associated with bacteriological confirmation (absent or present) and resistance profile of Mycobacterium tuberculosis (34.06 ± 9.272 IU/L versus 29.56 ± 7.917 IU/L; F = 9.875; *p* = 0.002) (Figure 1b), delayed diagnosis of PTB (34.70 ± 9.124 IU/L versus 28.60 ± 7.692 IU/L; F = 20.779; *p* = 0.000), and cavitary PTB, as an obvious outcome of late detection of active TB disease (34.17 ± 9.231 IU/L versus 30.11 ± 8.349 IU/L; F = 8.963; *p* = 0.003), with death as the final outcome (37.17 ± 9.937 IU/L) compared to survivors (32.39 ± 8.919) (F = 5.940; *p* = 0.016). Analysis of ADA plasmatic average levels among patients from group 1, with different patterns of drug-resistant or -sensitive TB, revealed equal distribution among subcategories of bacteriologically confirmed PTB (34.07 ± 9.31 IU/L in DS-TB, 37.00 ± 12.57 IU/L in DR-TB, 35.50 ± 7.14 IU/L in RR-TB and 31.29 ± 7.15 in MDR-TB) compared to clinically diagnosed PTB (29.56 ± 7.91) (Figure 1b).

Serum ADA levels increased proportionally with the severity of malnutrition as assessed by the Malnutrition Universal Screening Tool (MUST), rising from 28.91 ± 6.44 IU/L in patients with a MUST score of 1 to 35.07 ± 10.19 IU/L in those with a score of 5 (F = 2.487; *p* = 0.024; R = 0.154; R^2^ = 0.024; η = 0.263; η^2^ = 0.069). The highest risk of malnutrition, identified in 133 patients, was also significantly associated with elevated ADA levels (F = 3.378; *p* = 0.036; R = 0.153; R^2^ = 0.024; η = 0.179; η^2^ = 0.032).

Inflammatory tests had elevated values in group 1 (*p* = 0.000), except for CRP, which had a similar distribution of serum levels in both groups of patients (*p* = 0.998) (Table 2).

The mean count of leukocytes, neutrophils, and platelets registered higher values among patients from group 1, and, in contrast, lymphocytes and NLR were approximately equally distributed among group 1 and 2 (*p* = 0.934) (Table 2). PLR and SII had elevated plasmatic levels in patients from group 1 (*p* = 0.014 and *p* = 0.018, respectively) (Table 2). According to ANOVA test and measure of association, the poor nutritional status of patients, evaluated through underweighting and different inflammatory biomarkers, was associated with significant elevation in absolute counts of leukocytes (*p* = 0.002), neutrophils (*p* = 0.000), and platelets PLR (*p* = 0.000), ESR (*p* = 0.000), and SII (*p* = 0.000). Mean value of SII (1752.226 ± 2704.150) significantly increased not only in bacteriologically confirmed PTB cases (2006.477 ± 3028.184; F = 5.640; *p* = 0.018) (Table 2), but also in patients with delayed diagnosis of PTB (2115.317 ± 3096.085; F = 9.636; *p* = 0.002), cavitary PTB (2126.229 ± 3110.138; F = 9.535; *p* = 0.002), and deceased (3308.1625 ± 3023.074; F = 9.344; *p* = 0.003).

**Figure 2:**
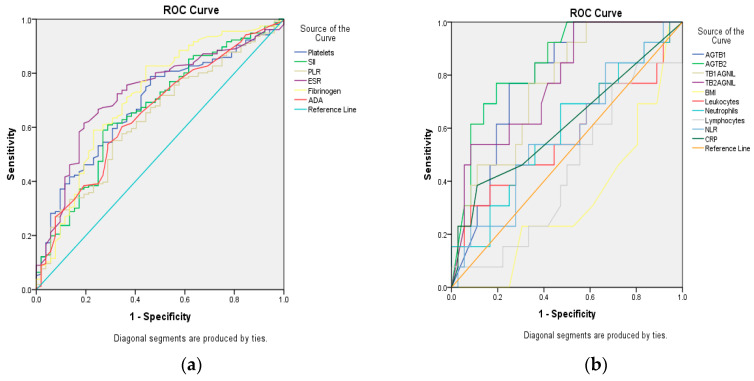
Receiver operating characteristic (ROC) curve analysis for assessment of the area under the curve (AUC) for biomarkers. (**a**) platelets; systemic immune inflammatory index (SII = absolute neutrophil count × absolute platelet count/absolute lymphocyte count); platelets/lymphocytes ratio (PLR); erythrocyte sedimentation rate (ESR); Fibrinogen, adenosine deaminase (ADA); (**b**) Antigen Tuberculosis 1 (AgTB1), Antigen Tuberculosis 2 (AgTB2), Tuberculosis 1 Antigen NIL (TB1AgNIL); Tuberculosis 2 Antigen NIL (TB2AgNIL); body mass index (BMI), Leukocytes; Neutrophils; Lymphocytes; NLR = neutrophils/lymphocytes ratio; C-reactive protein (CRP).

Both ADA and SII followed a similar pattern of elevated mean values in relation to PTB activity status and disease severity, including mortality. To further assess their diagnostic performance, receiver operating characteristic (ROC) curve analysis was conducted, with the area under the curve (AUC) used to identify cut-off values, sensitivity, and specificity for each biomarker (Figure 2). Only biomarkers with an AUC ≥ 0.6 were considered for interpretation (Table 3). The highest AUC was observed for AgTB2, which reached a value above 0.8 and yielded a cut-off of ≥6.23 IU/mL, with a sensitivity of 75% and specificity of 22%. Despite this performance, the concordance rate between positive IGRA results and positive bacteriological confirmation (smear and/or culture) was 31.70% (13 out of 41 cases), and IGRA testing was therefore not considered reliable for diagnosing active PTB in this cohort.

An AUC of 0.708, for a serum ADA cut-off value of 30.5 IU/L, had 75% sensitivity and 22% specificity. Values higher than 30.5 IU/L of serum ADA were reached by 116 patients (57.77%), with significant distribution among cases from group 1 and 2 [nG1 = 96/156; 61.53%% versus nG2 = 20/52; 38.46%; OR = 4.16; (2.26–7.64); RR = 2.21 (1.49–3.28); χ^2^ = 22.36; *p* = 0.000]. A level of ADA higher than 30.5 IU/L showed an 83% positive predictive value (PPV) and 35% negative predictive value (NPV) for mycobacterial activity. In predicting the risk of death, plasmatic levels of ADA greater than cut-off (75% sensitivity, 46% specificity) were reported in 18 deceased [n = 18/116; versus n = 6/92; OR = 2.63 (0.99–6.93); RR = 2.37 (0.98–5.75); χ^2^ = 4.048; *p* = 0.045].

For SII, ROC analysis revealed an AUC of 0.765, a cut-off equal to 902, with 50% sensitivity and 16% specificity. Higher values than the SII cut-off were found among 110 patients, with significant difference among groups [nG1 = 94/156; 60.25% versus n = 16/52; 30.77%; OR = 5.22 (1.71–15.87); RR = 4.45 (1.57–12.58); χ^2^ = 10.046; *p* = 0.002]. The sensitivity (83%) and specificity (51%) of SII in predicting death [n = 20/110; versus n = 4/98; OR = 5.22 (1.71–15.87); RR = 4.45 (1.57–12.58); χ^2^ = 10.046; *p* = 0.002] were higher than for differentiating the advanced forms of PTB with bacteriological positivity.

A high-risk subgroup was identified by applying threshold values for both ADA and SII, defined as serum levels exceeding their respective cut-off points. This combined ADA-SII profile was present in 73 patients. The prevalence of this high-risk profile was significantly higher in bacteriologically confirmed PTB cases (group 1: 63 out of 156 patients; 40.38%) compared to clinically diagnosed cases (group 2: 10 out of 52 patients; 19.23%), with statistically significant associations [OR = 2.84; 95% CI: 1.33–6.08; RR = 2.10; 95% CI: 1.16–3.78; χ^2^ = 7.62; *p* = 0.006]. Furthermore, the mortality rate within the high-risk ADA-SII subgroup was notably elevated (17 out of 73 patients; 23.28%) compared to those with ADA and SII values below the defined thresholds (7 out of 135 patients; 5.18%), again with strong statistical significance [OR = 5.55; 95% CI: 2.18–14.13; RR = 4.49; 95% CI: 1.95–10.32; χ^2^ = 15.138; *p* < 0.001].

## 4. Discussion

Tuberculosis continues to represent a major global health challenge, characterized by long-standing endemicity and recent exacerbation due to intersecting epidemics, notably HIV and COVID-19 [20,21,22,23]. Early diagnosis and prompt initiation of directly observed therapy remain essential strategies for limiting transmission, particularly from smear-positive individuals to their contacts. Nonetheless, conventional diagnostic criteria for pulmonary tuberculosis (PTB) present notable constraints in clinical settings. In TB cases, diagnostic certainty often remains elusive, despite supportive clinical symptoms or radiological abnormalities suggestive of active disease [24,25]. Subclinical and incipient forms of TB are inherently difficult to detect, often presenting with nonspecific or silent profiles that mimic post-infectious sequelae [25]. Current evidence on the utility of host- or pathogen-derived biomarkers in identifying active TB disease and improving diagnosis is inconclusive [26,27,28,29]. Pathogen biomarkers specific to LAM or DNA of MTB revealed epidemiological molecular tests using gene or antigen signatures [28]. In our study, all positive genotypic investigations of sputum or bronchial aspirate (such as LPA, GeneXpert MTB/RIF, and Xpert MTB/MDR) were associated with phenotypical positive smears and/or culture in bacteriologically confirmed PTB.

Over recent decades, TB research has prioritized pathogen detection strategies and pharmacological optimization of anti-tubercular regimens, while the identification of host immunological markers—whether adaptive, humoral, or cellular—has received comparatively limited attention. Cytokine and chemokine profiling, although relevant in differential diagnosis between TB infection and disease, is not routinely feasible in most clinical laboratories due to restricted access to technologies such as flow cytometry [28]. The validation of reliable host-derived blood biomarkers remains a complex and multifactorial undertaking.

Interferon-gamma release assays (IGRAs), particularly the QuantiFERON-TB Gold Plus (QFT), initially emerged as a promising diagnostic adjunct. However, their role has been refined over time, now largely confined to the detection of latent tuberculosis infection (LTBI) [30]. Nevertheless, the risk of progression to active TB in IGRA converters is non-negligible [31]. In our study, two patients with recent QFT conversion were subsequently diagnosed with clinically active PTB.

C-reactive protein (CRP), despite extensive investigation, has not demonstrated sufficient diagnostic performance to meet meta-analytical benchmarks for screening or risk stratification in TB [32].

Although plasmatic ADA has been spectrophotometrically determined since 1984, this enzyme is measured especially in fluids, being consecrated as an accurate diagnostic tool of pleural TB or other extrapulmonary TB forms such as meningitis, pericarditis, and peritonitis, with different cut-offs of 40 IU/L or 66 IU/L [33,34]. Recently, more studies proposed serum ADA level measurements as a useful marker for diagnosing cases of pulmonary TB when bacteriological confirmation is missing [8,35].

Adaptive immunity has a pivotal role in TB disease pathogenesis, with a strong and specific pro-inflammatory sequence, in balance with anti-inflammatory responses for controlling the TB process [36,37]. Based on this dynamic and complex inflammatory hallmark of TB pathogenesis, the assessment of host-blood inflammatory and immune biomarkers is recommended mainly for diagnosing and monitoring therapy [38]. It is time to cross the bridge from pleural space to plasma, with more research, larger cohorts, and longitudinal studies about the relevance of plasmatic ADA as a biomarker of TB disease and pattern recognition as an alternative method of diagnosing sputum negative PTB. In the last 2 decades, different cut-offs of ADA have been mentioned in the literature. Saini V et al. found a mean ADA of 31.107 ± 29.32 in TB patients with positive sputum and a much higher one (39.478 ± 32.22) among sputum negative TB patients [39], and Salmanzadeh S. et al. found a lower significant serum ADA (24 IU/L) for PTB, with 35% sensibility and 91% specificity [40]. There are findings to support high sensitivity versus others related to low sensitivity from 12% to 44% and higher specificity with a range above 96% in the validation of serum ADA [41,42]. According to our results indicating 75% sensitivity for serum ADA level, it can be considered an alternative tool for sputum negative PTB diagnosis, as another study revealed [42].

Serum ADA has the tendency to be at its peak in the first month of diagnosing PTB, decreasing to normal after one month of administered anti-TB therapy [42,43,44]. In our study, stratification cases, using PTB bacteriological confirmation status, revealed significant differences in the mean of ADA plasmatic levels between sputum positive and negative cases (*p* = 0.002) before anti-TB therapy was administered, highlighting higher values in bacteriologically confirmed PTB cases.

Systemic immune inflammatory index (SII) is a parameter like NLR or PLR, which is easy to calculate using a specific formula, depending on the counts of lymphocytes, neutrophils, and platelets. There is an interest in SII in cancer but fewer studies in tuberculosis and none in advanced forms of PTB. Kerget B et al., in their study on differential diagnosis between TB and sarcoidosis, found increased SII above 890.7 for an AUC of 0.668, with 70% sensitivity and 66% specificity, related to the concept of progressive inflammation and caseification necrosis, which explains the formation of TB granuloma [45] but not cavitation. In determining COVID mortality, a cut-off over 618.8 of SII was related to higher mortality [46]. In our study, the cut-off for SII was greater (but with lower sensitivity and specificity).

To the best of our knowledge, there is no previous study that assessed the predictive value of both ADA and systemic immune inflammatory index in advanced PTB. Although the reduced size of the cohort can be considered a limitation, the triangle between bacteriologically negative and positive PTB illness and deaths was significantly impacted by ADA and SII duet biomarker, with values greater than 30.5 IU/L, respectively 902. We recommend this duet be performed in TB patients, in their first week after admission to hospital, before anti-TB therapy is started, considering this duet as an early predictive biomarker of PTB severity.

Our study aimed to evaluate the prognostic performance of two inflammatory biomarkers—serum adenosine deaminase (ADA) and the systemic immune inflammatory index (SII)—in a cohort of adult patients with pulmonary tuberculosis (PTB) stratified by bacteriological confirmation, imaging features, nutritional status, and survival outcomes. Elevated values of both ADA and SII were found to be associated with parameters commonly indicative of severe PTB.

In our research, serum ADA showed a consistent pattern of elevation in association with key severity indicators. Mean levels were higher in patients with bacteriologically confirmed PTB (34.06 IU/L), delayed diagnosis (34.70 IU/L), cavitary lesions (34.17 IU/L), and fatal outcomes (37.17 IU/L), increasing progressively. SII showed a similar pattern of predicting severity, peaking in patients with fatal outcomes (3308.16) and remaining significantly elevated in other severe subgroups, including those with bacteriological confirmation (2006.48), delayed diagnosis (2115.32), and cavitary lesions (2126.23). These parallel escalations underscore a pattern in which both ADA and SII reflect disease burden.

When directly compared with the external dataset from Salmanzadeh et al. (2015), who reported a mean ADA of 26 IU/L in PTB and a specificity of 91% at this threshold [40], our findings present higher mean ADA values overall (32.94 ± 9.15 IU/L) and even greater elevations in high-risk groups. This discrepancy may reflect differences in cohort composition, disease severity, and regional epidemiology. The present cohort was characterized by a high proportion of bacteriologically confirmed, cavitary, and malnourished patients—clinical features likely to amplify systemic inflammation and ADA expression.

Alaarag et al. reported a diagnostic sensitivity of 95% and specificity of 86.7% for serum ADA at a threshold of 30.15 U/L [47], reinforcing its utility as a diagnostic tool in distinguishing TB from non-TB cases. However, their study applied a binary classification framework and did not explore intra-disease heterogeneity. In contrast, our study focused exclusively on patients with pulmonary TB and introduced stratification based on radiological severity. This approach enabled us to assess whether ADA levels not only support diagnosis but also reflect disease burden. Notably, we observed significantly elevated serum ADA levels in patients with severe radiologic patterns—particularly those with cavitary lesions—suggesting a potential gradient of ADA activity aligned with pulmonary tissue destruction and overall disease severity.

The study by Gencheva et al. (2020), conducted in Bulgaria, explored the diagnostic value of serum adenosine deaminase (ADA) in a cohort of 66 patients with inflammatory lung diseases, including 12 cases of pulmonary tuberculosis (PTB), reporting a mean ADA of 28.14 U/L in the PTB group [48]. However, ADA levels were also elevated in the pneumonia (30.64 U/L) and pleural effusion (37.59 U/L) group. Gencheva’s findings reinforce the biochemical responsiveness of ADA to pulmonary inflammation [48], but our data contextualize this response within a TB-specific framework. Our findings suggest that within a confirmed PTB population, ADA retains discriminatory value when applied in a prognostic rather than diagnostic context. The exclusion of other conditions that could provide confounding data further mitigates the risk of misclassifications.

The evolution of serum ADA throughout the TB treatment has also been researched. While Soedarsono et al. emphasized ADA’s decline following treatment initiation (due to responsiveness to bacterial load reduction during treatment), our data illustrate a gradient increase in baseline ADA with disease severity [44]. Both studies support the association between ADA levels and bacterial burden. Soedarsono et al. demonstrated that patients with AFB +3 had the highest ADA levels at baseline (34.04 ± 9.40 IU/L), which decreased with treatment. Similarly, in our dataset, patients with cavitary lesions—a radiologic correlate of high mycobacterial burden—also had significantly higher ADA levels (35.29 ± 9.88 IU/L). Moreover, ADA levels averaged 37.17 ± 9.94 IU/L in deceased patients—well above the baseline value in Soedarsono’s cohort (26.40 ± 9.61 IU/L)—suggesting that high pretreatment ADA may forecast poor prognosis [44]. Importantly, in our analysis, this increase in ADA was independent of pleural involvement and was seen in systemically ill patients with pulmonary involvement alone. Taken together, both studies underscore the biological plausibility and operational value of serum ADA in PTB. Soedarsono et al. emphasize ADA’s role in dynamic monitoring, while our study repositions it—particularly when paired with SII—as a candidate prognostic biomarker.

From a mechanistic standpoint, SII may reflect the immunological state of TB patients in ways that extend beyond simple inflammation. Elevated SII is driven by neutrophilia and thrombocytosis, both of which are common in active TB and mediated by IL-6 and other pro-inflammatory cytokines. Simultaneously, lymphopenia, often observed in severe or disseminated TB, contributes to the denominator of the SII equation, amplifying the index in high-risk individuals. As such, SII integrates both pro-inflammatory excess and adaptive immune suppression—two hallmarks of severe TB infection.

The systemic immune inflammation index (SII) has gained increasing traction as a surrogate marker of immune activation across both infectious and non-infectious conditions. In the context of pulmonary tuberculosis (PTB), SII emerges as a relevant biomarker in both our study and that of Yu et al. (2024), albeit through different clinical lenses—diagnostic refinement in their case [49], and prognostic stratification in ours. Yu et al. conducted a retrospective diagnostic study involving 2030 T-SPOT.TB-positive individuals, aiming to distinguish active PTB from non-tuberculous lung diseases (NTLDs). Their biomarker panel included SII, fibrinogen, and T-cell response indicators (ESAT-6, CFP-10). SII was significantly elevated in PTB patients, positively correlated with CRP levels and sputum smear grade. As a standalone marker, its discriminative capacity was modest (AUC = 0.612); however, in combination, particularly with fibrinogen and T-SPOT components, its diagnostic specificity improved substantially.

Despite methodological and conceptual differences, both datasets underscore the link between SII and mycobacterial burden. Yu et al. demonstrated an association between elevated SII and positive microbiological exam, while we observed a parallel trend, with higher SII values in patients with cavitary lesions and bacteriologically confirmed TB. Although our study did not include fibrinogen and Yu et al. did not assess ADA, the underlying inflammatory domains intersect. Fibrinogen reflects hepatic-phase inflammation mediated by IL-6, while ADA is secreted by monocyte–macrophage lineages, in response to mycobacterial antigens. Both markers, when considered alongside SII, may act as converging co-signals of disease activity. This convergence reinforces the notion that SII could serve as a microbiological surrogate even in settings where molecular diagnostics are not readily available. While ADA has traditionally been linked to pleural TB, SII appears to capture systemic immune activation more broadly, independent of anatomical compartmentalization.

Our study further aligns with the findings of Ștefănescu et al. (2021), who examined a broader panel of hematologic parameters in a Romanian PTB cohort [50]. Though the marker panels differ, the underlying theme persists: systemic inflammatory markers carry meaningful clinical signals in TB, and their integration into risk models may enhance stratification beyond binary diagnostic endpoints.

Interestingly, even among patients who remained culture-positive after 2 months of therapy SII declined by ~50%, suggesting that it may reflect a partial microbiological response or systemic dampening of inflammation independent of complete sputum sterilization [50]. This raises the possibility that SII kinetics—rather than absolute values—may offer additional prognostic value, particularly when measured at multiple timepoints across treatment. Overall, the convergence of data between the two studies—conducted in distinct yet demographically similar Romanian cohorts—supports the reproducibility of SII as a marker of inflammatory status in PTB.

The parallelism in SII behavior across both cohorts—despite differences in biomarker scope and statistical modeling—suggests that this index may serve as a robust surrogate of disease burden and inflammatory resolution. Notably, both studies excluded patients with significant comorbidities or HIV, minimizing confounding factors from other sources of systemic inflammation. This enhances the specificity of SII as a TB-related biomarker within both datasets. While our study additionally included ADA as a parameter—given its role in T-cell-mediated immune responses in TB—the inflammatory ratios alone (especially SII) showed parallel behavior in both datasets.

Dong et al. provide a complementary—and equally compelling—view of systemic immune inflammatory indices by shifting the timeframe from baseline risk to treatment-related damage regarding 2.643 Chinese PTB patients treated between 2018 and 2023 [51]. The study propensity-matched 258 cases of drug-induced liver injury (DILI) to 258 controls, all of them hepatitis-B-surface-antigen positive. Their baseline sampling occurred two months after treatment initiation. The mean SII in the DILI group was 1 731 (SD 2 351), and the optimum prognostic boundary was slightly higher than ours, at 1 166 (AUC 0.668, 95% CI 0.614–0.721). At that cut-off, SII predicted emergent hepatotoxicity with 46% sensitivity and 82% specificity. However, the Chinese team improved discrimination (AUC 0.817) by combining SII with neutrophil-to-lymphocyte ratio (cut-off 2.93), monocyte-to-lymphocyte ratio (0.36), eosinophil proportion (1.78%), and the CD4/CD8 ratio (1.37). In their multivariable model, every unit increase in SII (×10^−3^) raised the odds of DILI by 0.1%, whereas a one-decile rise in EOS% increased the risk by 27%. Taken together, the two studies trace a coherent arc across the tuberculosis care pathway. At diagnosis—when our patients had not yet received hepatotoxic drugs—an ADA ≥ 30 IU L combined with an SII ≥ 902 signaled bacterial burden severe enough to double the prevalence of cavitation and to quadruple early mortality. Dong et al. found that, two months into treatment, an SII above about 1100, especially when accompanied by an NLR above 2.9 or an eosinophil fraction above 1.8%, heralded incipient liver failure in a hepatitis-B-coinfected population [51]. Thus, the same composite index, anchored on neutrophil, platelet, and lymphocyte counts, appears to mark vulnerability first to pulmonary tissue destruction and later to immune-mediated hepatocellular damage. These quantitative parallels strengthen the biological argument for routine use of SII—but supplemented by context-specific partners.

A relevant biomarker signature gains performance attributes when it has an AUC > 0.9 for all groups of investigated cases, with a sensitivity near 90% and a high specificity (above 80%) [52]. Although in our study neither serum ADA nor SII independently reached diagnostic specificity thresholds required for screening applications, their combined application yielded improved predictive power regarding the severe evolution of PTB (*p* = 0.000).

Furthermore, our data advance previous findings by integrating ADA with SII, a composite marker of immune response that encompasses neutrophil, platelet, and lymphocyte dynamics. The synergy of ADA and SII forms a dual biomarker signature—referred to here as a “biomarker duet”—which identifies a high-risk subgroup: patients exceeding the cut-offs of ADA > 30 IU/L and SII > 902, with fourfold greater odds of mortality and double the risk of advanced disease.

Within this subgroup, the mortality rate was markedly elevated (23.28%), and the proportion of bacteriologically confirmed PTB cases was significantly higher. The odds ratios and risk ratios associated with this biomarker duet underscore its potential to delineate patients with greater likelihood of severe or fatal disease, suggesting that the ADA-SII profile reflects both biological and clinical severity. Thus, beyond absolute serum ADA values, our integration of SII brings multiple aspects to the analysis. While ADA is anchored in lymphocytic activation, SII captures the broader interplay of neutrophilia, thrombocytosis, and relative lymphopenia—hallmarks of TB-induced immune dysregulation.

The combined assessment of ADA and SII delineates a clinically meaningful biomarker duet with relevance for prognostication in PTB. While neither marker achieves standalone diagnostic precision, their concurrent elevation is associated with classical features of advanced disease. Given their accessibility and cost-effectiveness, these biomarkers may be particularly useful in resource-limited settings or where microbiological confirmation is delayed.

However, the monocentric nature of the study limits external validity, and further multicenter validation is necessary. Future studies should explore external validation of the ADA-SII composite signature, potentially integrating it with imaging, molecular diagnostics, and clinical scoring systems to enhance precision. Prospective implementation in various clinical settings—including outpatient care, HIV co-infection, and pediatric populations—may broaden its applicability. Nonetheless, the present data highlight the potential of ADA and SII to serve as adjunctive tools in clinical decision-making, especially for early identification of high-risk patients requiring intensified monitoring or intervention.

## 5. Conclusions

Our results indicate that serum adenosine deaminase (ADA) and the systemic immune inflammatory index (SII) are associated with key indicators of disease severity in pulmonary tuberculosis (PTB), including advanced cavitary pulmonary lesions, bacteriological confirmation, diagnostic delay, and early mortality. Mean ADA concentrations were notably elevated in fatal cases (37.17 ± 9.94 IU/L) relative to the cohort average (32.94 ± 9.15 IU/L), while SII values reached their highest levels in the same subgroup (3308.16 ± 3023.07), suggesting their potential value in early risk stratification.

A high-risk subgroup defined by ADA > 30 IU/L and SII > 902 was identified as bearing a significantly higher burden of disease. This high-risk subgroup exhibited a fourfold increased risk of mortality and a twofold greater likelihood of presenting with advanced PTB, with strong representation among bacteriologically confirmed cases.

These findings support the application of ADA and SII—particularly when assessed jointly—as accessible and potentially cost-effective biomarkers for risk stratification in PTB. Their utility may be most pronounced in resource-constrained contexts or in clinical scenarios where definitive microbiological confirmation remains unavailable or delayed. Validation in independent, multicentric cohorts is warranted to determine the reproducibility and external applicability of these thresholds, particularly in TB-endemic regions.

## Figures and Tables

**Table 1:** Descriptive basic characteristics of pulmonary tuberculosis (PTB) patients.

Variable	Bacteriologically Confirmed PTB	Clinically Diagnosed PTB	Total Cases	*p*
Patients (n; %)	156 (75%)	52 (25%)	208 (100%)	
PTB Notified Cases	156	52	208	0.000
New cases (n; %)	106 (67.95%)	50 (96.15%)	156 (75%)	
Relapses (n; %)	50 (32.05%)	2 (3.85%)	52 (25%)	
Delayed diagnosis (n; %)	138 (88.46%)	10 (19.23%)	148 (71.15%)	0.000
Interval from onset of symptoms to PTB diagnosis (months)	3.55 ± 2.908	2.54 ± 2.601	3.30 ± 2.862	0.027
Deceased (n)	22 (14.10%)	2 (3.84%)	24 (11.53%)	0.046
Interval PTB diagnosis–death (mean) (days)	54.32 ± 76.61	8.50 ± 6.36	50.50 ± 4.35	0.416
Age at the moment of PTB diagnosis (years)	52.81 ± 14.13	59.04 ± 14.18	54.4 ± 14.40	0.007
Age at the moment of death (years)	56.36 ± 15.38	73.50 ± 10.60	57.79 ± 15.63	0.141
Gender (n; %)				0.203
M (males)	119 (76.28%)	35 (67.07%)	154 (74%)	
F (females)	37 (23.72%)	17 (32.93%)	54 (26%)	
Residence (Urban) (n; %)	78 (50%)	35 (67.30%)	113 (54.32%)	0.031
Smoking exposure (n)	122 (78.2%)	34 (65.38%)	156 (75%)	0.003
Current smokers (CS)	99	19	118	
Former smokers (FS)	23	15	38	
Never smokers (NS)	34	18	52	
Cigars pack-year (mean)	30.31 ± 15.61	37.21 ± 24.64	31.81 ± 18.10	0.366
Current smokers (CS)	31.39 ± 15.33	29.37 ± 18.15	31.07 ± 15.75	
Former smokers (FS)	25.65 ± 16.32	47.13 ± 28.68	34.13 ± 24.11	
Alcohol abuse use (n; %)	44 (28.20%)	10 (19.23%)	54 (26%)	0.340
Previous COVID-19 (n; %)	45 (28.85%)	31 (59.61%)	76 (36.53%)	0.000
Interval COVID–PTB (mean) (months)	10.12 ± 8.05	13.74 ± 9.55	11.57 ± 8.78	0.095
Height (cm) (mean)	170.28 ± 8.176	168.81 ± 8.67	169.91 ± 8.3	0.269
Weight (kg) (mean)	58.78 ± 13.19	67.87 ± 17.17	61.05 ± 17.78	0.000
Loss in weight (kg) (mean)	6.55 ± 5.69	2.79 ± 4.667	5.61 ± 5.68	0.000
BMI (kg/m^2^) (mean)	20.02 ± 4.01	24.02 ± 6.08	21.02 ± 4.92	0.000
Underweighting (BMI ˂ 18.5 kg/m^2^) (n; %)	70 (44.87%)	10 (19.23%)	80 (38.46%)	0.001
Malnutrition Universal Screening Tool (MUST) Score				0.000
0 (n; %)	36 (23.08%)	27 (51.92%)	63 (30.29)	
1 (n; %)	5 (3.21%)	6 (11.54%)	11 (52.88%)	
2 (n; %)	6 (3.85%)	2 (3.84%)	8 (3.85%)	
3 (n; %)	16 (10.25%)	2 (3.84%)	18 (8.65%)	
4 (n; %)	18 (11.54%)	4 (7.69%)	22 (10.57%)	
5 (n; %)	23 (14.74%)	7 (13.46%)	30 (14.42%)	
6 (n; %)	52 (33.33%)	4 (7.69%)	56 (26.92%)	
Risk of malnutrition by MUST				0.000
Low (n; %)	37 (23.72%)	27 (51.92%)	64 (30.77%)	
Medium (n; %)	5 (3.21%)	6 (11.54%)	11 (5.29%)	
Higher (n; %)	114 (73.07%)	19 (36.54%)	133 (63.94%)	
Nodular lung lesions (n; %)	153 (98.07%)	47 (90.38%)	200 (96.15%)	0.012
Cavitary lung lesions (n; %)	139 (89.10%)	6 (11.54%)	145 (69.71%)	0.000
Pulmonary miliary (n; %)	6 (3.84%)	3 (5.76%)	9 (4.32%)	0.6
Bronchopneumonia (n; %)	17 (10.89%)	1 (1.9%)	18 (8.65%)	0.005

Legend: PTB = pulmonary tuberculosis; BMI = body mass index; MUST = Malnutrition Universal Screening Tool.

**Table 2:** Baseline biomarkers′ characteristics in pulmonary tuberculosis (PTB) patients.

	Bacteriologically Confirmed PTB	Clinically Diagnosed PTB	Total Cases	*p*
ADA (mean) (IU/L)	34.06 ± 9.272	29.56 ± 7.917	32.94 ± 9.14	0.002
DS-TB (n = 139)	34.07 ± 9.31			
DR-TB (n = 6)	37.00 ± 12.57			
RR-TB (n = 4)	35.50 ± 7.14			
MDR-TB (n = 7)	31.29 ± 7.15			
QuantiFERON TB Gold Plus (n; %)	13 (8.33%)	38 (73%)	51 (24.51%)	
Positive results (nr; %)	13 (100%)	30 (78.94%)	43 (84.31%)	0.000
AgTB1 (IU/mL)	7.280 ± 2.351	3.794 ± 3.427	5.742 ± 3.09	0.000
AgTB2 (IU/mL)	7.317 ± 2.390	3.381 ± 3.083	5.392 ± 2.98	0.001
TB1AgNIL (IU/mL)	6.097 ± 2.989	3.187 ± 2.942	4.823 ± 2.92	0.004
TB2AgNIL (IU/mL)	6.268 ± 3.006	3.006 ± 2.838	4.718 ± 2.94	0.001
Leukocytes (mean) {cells}/µL	11,169.49 ± 5114.582	8845.58 ± 2672.459	10,588.51 ± 4729.138	0.002
Neutrophils (mean) {cells}/µL	7815.46 ± 4892.382	5792.48 ± 2564.178	7309.71 ± 4507.062	0.005
Lymphocytes (mean) {cells}/µL	2101.99 ± 1033.384	2089.08 ± 756.555	2098.76 ± 969.884	0.934
Platelets (mean) {cells}/µL	36,0064.10 ± 16,6002.358	268,250.00 ± 110,328.542	337,110.58 ± 158,812.873	0.000
NLR (mean)	6.20 ± 16.18	5.08 ± 11.67	5.92 ± 15.16	0.648
PLR (mean)	215.63 ± 169.08	153.17 ± 109.91	200.02+/158.49	0.014
SII (mean)	2006.47 ± 3028.18	989.47 ± 1009.76	1752.22 ± 2704.15	0.018
ESR (mean) mm/hour	59.68 ± 32.03	33.98 ± 26.74	53.25 ± 32.69	0.000
CRP (mean) mg/dL	50.76 ± 81.65	50.82 ± 154.46	50.78 ± 105.09	0.998
Fibrinogen (mean) (g/L)	5.23 ± 1.76	4.19 ± 1.38	4.97 ± 1.73	0.000

Legend: PTB = pulmonary tuberculosis; ADA = adenosine deaminase; DS-TB = drug-sensitive tuberculosis; DR-TB = drug-resistant tuberculosis; RR-TB = rifampicin-resistant tuberculosis; MDR-TB = multidrug-resistant tuberculosis; TB = tuberculosis; Ag = antigen; NLR = neutrophils/lymphocytes ratio; PLR = platelets/lymphocytes ratio; SII = systemic immune inflammatory index (absolute neutrophil count × absolute platelet count/absolute lymphocyte count); ESR = erythrocyte sedimentation rate; CRP = C-reactive protein.

**Table 3:** ROC analysis of biomarkers′ cut-off value in predicting the risk of death among pulmonary tuberculosis patients.

Biomarker	AUC	Std. Error	*p*	Asymptotic 95% CI		Coordinates of the ROC Curves		
				Lower Bound	Upper Bound	Cut-off Value	Sensitivity	Specificity
CRP	0.856	0.043	0.000	0.772	0.939	72	0.64	0.15
NLR	0.843	0.070	0.000	0.707	0.980	4.73	0.36	0.22
Neutrophils	0.805	0.062	0.000	0.684	0.926	7480	0.36	0.26
SII	0.765	0.048	0.000	0.670	0.860	902.14	0.50	0.16
Leukocytes	0.744	0.075	0.002	0.596	0.891	11350	0.39	0.18
ADA	0.708	0.094	0.032	0.524	0.892	30.5	0.75	0.22
PLR	0.706	0.058	0.001	0.593	0.820	175.88	0.42	0.14
ESR	0.739	0.043	0.000	0.654	0.824	77.00	0.58	0.25
Platelets	0.659	0.115	0.103	0.433	0.885	365.00	0.50	0.03
Fibrinogen	0.657	0.093	0.105	0.475	0.839	3.85	0.67	0.39
BMI	0.334	0.074	0.039	0.190	0.479	-	-	-
Lymphocytes	0.306	0.090	0.016	0.130	0.481	-	-	-

Legend: AUC = area under the curve; ADA = adenosine deaminase; NLR = neutrophils/lymphocytes ratio; PLR = platelets/lymphocytes ratio; SII = systemic immune inflammatory index (absolute neutrophil count × absolute platelet count/absolute lymphocyte count); ESR = erythrocyte sedimentation rate; CRP = C-reactive protein; BMI = body mass index; null hypothesis: true area = 0.5.

## Data Availability

Data not included in a public database.

## Abbreviations

The following abbreviations are used in this manuscript:

ADA	Adenosine Deaminase
TB	Tuberculosis
PTB	Pulmonary Tuberculosis
SII	Systemic Immune Inflammatory Index
MTB	Mycobacterium tuberculosis
WHO	World Health Organization
CT	Computed Tomography
TST	Tuberculin Skin Test
IGRA	Interferon Gamma Releasing Assay
BMI	Body Mass Index
MUST	Malnutrition Universal Screening Tool
LPA	Line Probe Assay
NLR	Neutrophil-to-Lymphocyte Ratio
PLR	Platelet-to-Lymphocyte Ratio
ESR	Erythrocyte Sedimentation Rate
CRP	C-Reactive Protein
QFT	QuantiFERON-TB Gold Plus
ROC	Receiver Operating Characteristic
AUC	Area Under the Curve
PPV	Positive Predictive Value
NPV	Negative Predictive Value
DR-TB	Drug-Resistant Tuberculosis
RR-TB	Rifampicin-Resistant Tuberculosis
MDR-TB	Multidrug-Resistant Tuberculosis
DS-TB	Drug-Sensitive Tuberculosis
